# Low-cost embedded system for spectral power distribution reconstruction for controlled environmental agriculture using a multispectral sensor and cloud-based deep learning

**DOI:** 10.1016/j.ohx.2026.e00786

**Published:** 2026-05-07

**Authors:** Juan Morales-Guerra, Juan Soto-Perdomo, Juan Botero-Valencia, Erick Reyes-Vera, J.M. Pearce

**Affiliations:** a Faculty of Engineering, Instituto Tecnológico Metropolitano, Grupo SCR, Medellín 050034, Colombia; b Faculty of Engineering, Instituto Tecnológico Metropolitano, Grupo AEYCC, Medellín 050034, Colombia; c Department of Electrical & Computer Engineering and Ivey Business School, Western University, London, ON, Canada

**Keywords:** Cloud computing, Low-cost hardware, Spectral power distribution, Spectral reconstruction, Agriculture monitoring, Controlled environmental agriculture

## Abstract

This work presents an open-source device for acquiring, correcting, and reconstructing the spectral power distribution (SPD) of LED sources used in controlled environmental agriculture. Unlike direct measurement spectrometers, the system employs a low-cost multispectral sensor (AS7265x, 18 channels, 410–940 nm) to acquire sparse band-integrated data, which are subsequently processed through a two-stage machine learning pipeline to infer a dense SPD representation. The sensor is integrated into an embedded platform that performs spectral acquisition, processing, wireless transmission, and remote visualization. Comparison with a reference spectrometer revealed non-linearities and some minor limits to the agreement between sensor data and ground-truth spectra. To address this, a correction stage based on a multilayer perceptron (MLP) implemented with TensorFlow Lite Micro was developed, reducing the RMSE from 0.183 to 0.035 and improving the reliability of the data. Complementary environmental monitoring was included using a BME688 sensor to record temperature, humidity, and gas concentration, serving as a reference to detect and correlate anomalies in SPD measurements under extreme environmental conditions. All data were transmitted to a back-end server for processing. Spectral reconstruction was performed in the cloud using a one-dimensional convolutional neural network (1D-CNN) trained on horticultural LED spectra and physically inspired synthetic spectra representative of CEA. The model achieved an RMSE of 0.0135, confirming high precision within the target application domain and demonstrating a scalable and cost-effective solution for spectral monitoring in controlled agricultural environments.

## Graphical abstract

**Figure:**
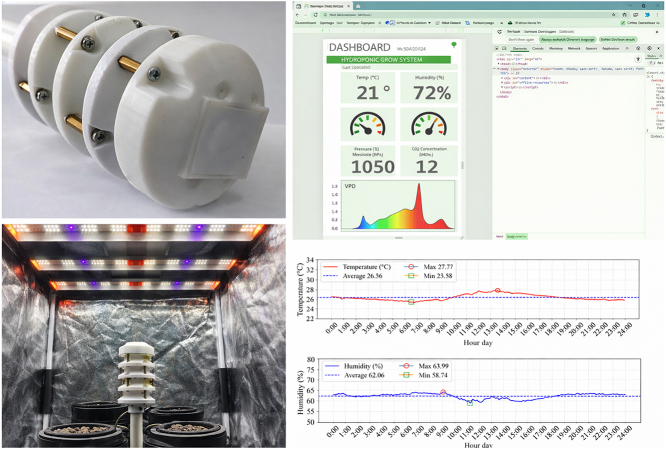

**Table:** 

**Specifications table**	

Hardware name	Spectral sensing device

Subject area	• Engineering • Instrumentation • Spectroscopy • Environmental monitoring • Internet of Things (IoT)
Hardware type	• Measuring physical properties and in-lab sensors • Field measurements and sensors • Electrical engineering and computer science
Open source license (hardware)	CERN OHL v2-S
Open source license (documentation)	Creative Commons Attribution-ShareAlike (CC BY-SA)
Cost of hardware (prototype)	US$144.14
Closest commercial alternative	HPCS320 Portable Spectrometer ($AÃ500–600)
Source file repository	https://doi.org/10.17605/OSF.IO/UM62Q

	

## 1 Hardware in context

Controlled-environment agriculture (CEA) has emerged as a viable alternative to open-air agriculture because it enables precise management of environmental factors such as temperature, humidity, nutrients, CO${}_{2}$, and light spectrum [1], [2], [3], [4], [5]. Recent studies have shown that both spectral composition and irradiance of light sources have a significant impact on plant morphology [6], photosynthetic efficiency [7], yield [8], pigment synthesis [9], [10], and the production of secondary metabolites [11], [12]. Studies on leafy vegetables, herbs, and fruit crops have shown that even subtle changes in spectral composition or intensity of artificial lighting can significantly affect growth rate [13], biomass accumulation [14], nutritional content [15], and overall resource use efficiency [16]. As a result, the capacity to monitor and adjust SPD in real time has become critical not only for plant physiology research but also for lighting optimization and energy-efficient operation in indoor farms [17]. In addition, the photoperiod plays a major role in economic viability, as lighting can account for a major share of operating costs in indoor farms [18], [19].

Conventional laboratory-grade spectroradiometers, such as Czerny-Turner field and fiber-coupled instruments, offer subnanometer spectral resolution and are commonly used as reference tools for optical measurements. However, they are expensive (typically $2000 to $6000), fragile, and poorly suited for long-term deployment in greenhouses and vertical farming systems. Table 1 summarizes some commercially available spectroradiometers, including the Apogee MS-100, AvaSpec-ULS2048CL-EVO, and Apogee PS-200. These devices offer excellent performance, but they remain costly and sensitive to environmental factors, limiting their suitability for continuous monitoring in humid or dust-prone agricultural environments.

Previous work on open hardware [20], [21] has demonstrated massive reductions in economic costs for scientific equipment [22], [23], [24], normally about ten percent of the cost of proprietary equipment [25]. Cost reductions improve accessibility of the equipment for under-resourced labs [26].

Following this open hardware model, a new generation of low-cost, open-hardware spectrometers have emerged, combining multichannel optical sensors, embedded computing, artificial intelligence-assisted processing, and additive manufacturing [3], [27], [28], [29], [30], [31]. For example, Rahman et al. presented an open-source photosynthetically active radiation (PAR) sensor based on the AMS AS7341, which achieves 1–5% error compared to a reference Apogee SQ-500SS at a fraction of the cost [3]. Similarly, Botero et al. introduced a portable IoT multispectral sensor that combines a Particle Photon MCU with AMS AS7262 and AS7263 modules to sample twelve narrow bands between 450 and 860 nm [28]. The battery-powered device, housed in a 3D-printed PLA enclosure with PTFE and glass diffusers, lasts up to 24 h and includes a TinyML-optimized multilayer perceptron (MLP) for real-time spectral reconstruction. After normalizing the twelve raw channel intensities, the MLP, trained offline against reference spectra acquired from an AQ6373 spectrometer, corrects sensor biases and interpolates a continuous SPD across the 400–800 nm range with less than 2% RMSE, while using only mW of power on the MCU. Likewise, Fernández-Alonso et al. introduced a seven-band, LED-cycled reflectance spectrophotometer built on Arduino Mega in 2023 [29]. This device combines narrowband LEDs (400–800 nm), synchronous demodulation, and adaptive amplification, achieving 0.4–1.4% reflectance accuracy and 0.61% NDVI precision. In the same year, Myland et al. leveraged convolutional neural networks (CNNs) to reconstruct absolute SPD from a ten-channel AS7341 sensor [32]. Trained on real-world lighting scenarios and normalized to absolute irradiance, their 1D-CNN achieved a normalized RMSE below 1.6, highlighting the potential of deep learning for recovering dense spectra from sparse multispectral measurements.

In early 2024, García et al. demonstrated that a Hamamatsu C12880MA micro-spectrometer can be paired with a Raspberry Pi to perform on-demand fluorescence, absorbance, and scattering measurements in the field [30]. Their compact, fiber-optic design captures light via a bifurcated probe, digitizes spectra on board, and streams data for immediate analysis, yet its reliance on delicate fiber coupling and the single-pixel sensor limits spatial sampling. Later that year, Van Hoorn et al. took the opposite tack. Instead of focusing light into a spectrometer, they sequentially illuminated an entire scene with 64 narrow-band LEDs across eight selected wavelengths and captured each LED pulse with a global-shutter monochrome camera (Arducam OV2311) [37]. By cycling through all LEDs in under a second, they reconstructed a full multispectral image while maintaining high spatial resolution. In addition, Barjaktarović et al. combined four OV9782 imaging sensors with a Seek Mosaic Core thermal module on Raspberry Pi 4 [38]. A Fabry–Pérot filter array yields nine narrow bands; a two-step alignment pipeline delivers R^2^ = 0.986 against hyperspectral ground truth. In addition, Botero et al. built a dual-face spectrometer for leaves using two nine-band detectors (top and bottom) within a 3D-printed chassis [27]. Each detector alternately measures reflectance and transmittance under digitally controlled LEDs, capturing 410–915 nm at both interfaces. A color-checker calibration and an on-board TinyML MLP yield real-time corrected spectra with minimal power draw, enabling rapid, dual-side plant diagnostics in a handheld form factor. Finally, Lopin et al. conducted a comprehensive evaluation of low-cost AMS sensors (AS7262, AS7263, AS7265x) for nondestructive chlorophyll estimation across five leaf species, achieving R^2^ values up to 0.96 and MAE of 3.5 μg/cm^2^ after applying robust calibration and outlier rejection strategies [31].

**Table 1:** Market survey of representative laboratory-grade spectroradiometers.

Model/Manufacturer	Spectral range (nm)	Resolution (FWHM)	Approx. cost (USD)
Apogee MS-100 [33]	380–780	10 nm	$2199
AvaSpec-ULS2048CL [34]	200–1100	0.06–20 nm	$4840
Apogee PS-200 [35]	300–850	0.85 nm	$6174.85
XL-500A BLE [36]	340–1010	10 nm	$828.00

Beyond hardware miniaturization, recent studies have also explored computational approaches to recover or predict dense spectral information from limited observations. In particular, spectral reconstruction from sparse multispectral measurements has emerged as a relevant strategy when the sensor output is insufficient to fully represent the underlying spectral distribution. This issue is especially important in CEA, where discrete-channel sensors may capture general spectral tendencies but may not fully resolve narrow horticultural LED peaks, mixed blue–red spectral distributions, or band-integrated quantities relevant to photosynthesis and photomorphogenesis. Therefore, spectral reconstruction is not merely a post-processing enhancement but a necessary step to transform sparse low-cost sensor measurements into a denser SPD representation that is more informative, more comparable to reference spectroradiometers, and more suitable for lighting analysis and optimization [32].

A related but distinct line of research has focused on predicting or modeling LED spectral behavior from source operating conditions rather than reconstructing spectra from external low-cost sensor measurements. For example, Fan et al. proposed a machine-learning-assisted method to predict the SPD of full-spectrum white LEDs by combining Gaussian spectral decomposition with neural networks driven by electrical and thermal variables [39]. In subsequent work, Fan et al. introduced a thermal-electrical-spectral model to dynamically predict the optical and chromatic behavior of LED arrays under realistic operating conditions [40]. Additional studies have analyzed luminous flux degradation, SPD evolution, and electro-photo-thermal behavior in LEDs using response-surface models, Gaussian-based SPD methods, and physics-based electro-thermal formulations [41], [42], [43], [44]. Although these studies do not address inverse spectral sensing from low-cost multispectral observations, they demonstrate that computational modeling is an effective route to recover spectrally meaningful information when direct high-resolution measurements are limited or impractical.

Despite these valuable advances, all of the above systems still present limitations that restrict their scalability and long-term implementation in CEA. First, most of the proposed solutions rely on a limited number of spectral channels, typically between 6 and 12, which constrains their ability to capture fine spectral structure and near-infrared information [3], [28], [29], [37]. This limitation is particularly relevant for photosynthetic and morphogenetic responses. Furthermore, none of these designs integrates sensors to simultaneously monitor environmental parameters such as temperature, humidity, and volatile organic compounds, factors that strongly modulate plant physiology and spectral signatures. These shortcomings motivate the development of a unified and robust platform capable of extending spectral coverage, integrating environmental sensing, and combining embedded and cloud-based learning for reliable and scalable monitoring in controlled agriculture.

Previous studies have consistently highlighted that, although the AS7265x sensor provides a broad spectral range, calibration remains one of the main challenges to obtaining reliable results. In agricultural authenticity and classification tasks, Masithoh emphasized the need for pre-calibration using white and dark references, along with conversion to absorbance, to improve sample separability [45]. Similarly, Al and collaborators addressed consistency issues by controlling geometry and standardizing integration time and measurement distance [46]. In poultry freshness detection, Feyissa corrected spectral biases with the support of laboratory-grade spectrometers as a Ref. [47]. Palma, in contrast, applied a systematic bias-correction model to align AS7265x readings with laboratory equipment [48]. In a microfluidic biosensor, Bai relied on calibration curves built from standard bacterial concentrations [49]. For photosynthetically active radiation (PAR) estimation, Rahman and Jamil employed reference-based calibration, in addition to spectral normalization and empirical factors to compensate for cosine-response limitations [3], [50]. Henderson, when combining the AS7265x with imaging systems, had to correct spectral shift and cross-talk artifacts [51], whereas Sutherland struggled to distinguish subtle physiological states in lobsters even after exhaustive calibration efforts [52]. Sulistyo overcame sugar adulteration detection challenges by training neural networks on reference-calibrated mixtures [53], while Mohammed linked calibrated spectra with destructive quality parameters of fruits to predict shelf life under controlled storage [54]. Altogether, these works demonstrate that the success of the AS7265x critically depends on application-specific calibration strategies.

Unlike a general-purpose spectrometer, which directly resolves spectral content through optical dispersion or filtering, the present system performs spectral reconstruction from 18 discrete band-integrated measurements provided by the AS7265x sensor. The cloud-based CNN-1D infers a dense 411-point SPD from these measurements using learned statistical priors; therefore, the output should be interpreted as a model-predicted spectrum rather than as a direct physical measurement. Consequently, reconstruction accuracy depends on the consistency between the measured source and the model training distribution.

This work presents a low-cost and fully open-source platform that overcomes key limitations identified in previous multispectral sensing systems for controlled environmental agriculture. Unlike earlier open-hardware prototypes restricted by a narrow number of channels, fragile optical assemblies, or the lack of integrated environmental context, the proposed design employs the full 18-band AS7265x array to extend spectral coverage from the visible to the near-infrared region, capturing both photosynthetically active radiation and morphogenetically relevant wavelengths that govern plant development. Simultaneously, a BME688 module provides continuous measurements of temperature, humidity, pressure, and volatile organic compounds, allowing spectral data to be interpreted under real environmental conditions. A hybrid edge–cloud architecture combines local intelligence with scalable cloud processing: a TinyML MLP deployed on the ESP32-S2 performs on-device correction and normalization of the raw spectral data, while a cloud-hosted 1D-CNN reconstructs dense spectral distributions at 1 nm resolution. This dual-stage pipeline mitigates the nonlinearities and limited resolution inherent to the low-cost sensor. The reconstruction model was trained on a representative dataset of horticultural LED spectra and simulated spectral variations relevant to CEA. Accordingly, the model is expected to perform reliably within its training distribution, while sources outside this domain – including broadband phosphor-converted white LEDs, multi-peak composite sources, or spectrally shifted degraded LEDs – fall outside the validated scope and cannot be assumed to yield accurate reconstructions.

## 2 Hardware description

The developed open hardware integrates three complementary domains that together form the sensing system. At the electronic level, it combines the (1) AS7265x multispectral sensor, (2) the BME688 environmental sensor, and (3) the ESP32-S2 control board, which are responsible for data acquisition and initial preprocessing. From the mechanical perspective, the system relies on a modular enclosure fabricated by RepRap-class open-source fused filament fabrication-based 3D printing [55], [56], [57] with acrylic styrene acrylonitrile (ASA) filament, designed to protect the electronics, ensure optical alignment through a PTFE diffuser, and enable reproducible assembly. Finally, the software and IoT domain includes the embedded firmware with a TinyML MLP model for edge correction, and a cloud backend that runs a 1D CNN for spectral reconstruction, complemented by a web dashboard for real-time visualization and data querying. Together, these domains provide a modular, open, and scalable architecture aimed at spectral monitoring in controlled agricultural environments.

### 2.1 Design files and components

The proposed system integrates two complementary sensing modules that together provide both spectral and environmental information at the point of measurement.

The AS7265x multispectral sensor is a compact optical module that measures light intensity across eighteen different spectral bands in the visible and near-infrared ranges (410–940 nm) [58]. It consists of three stacked sensor dies – AS72651, AS72652, and AS72653 – each of which has six Fabry-Pérot interference filters with a FWHM of only 20 nm. The channels are centered at approximately 410, 435, 460, 485, 510, 535, 560, 585, 610, 645, 680, 705, 730, 760, 810, 860, 900, and 940 nm, covering the spectral regions directly related with photosynthetically active radiation (PAR), far-red responses, and near-infrared fingerprints. This device enables precise measurements of plant pigments, chlorophyll activity, and the spectrum composition of artificial lighting.

The BME688 continuously monitors key environmental variables, measuring temperature from −40 °C to 85 °C with an accuracy of ±0.5 °C, relative humidity from 0 to 100 %RH with ±3 %RH accuracy, and barometric pressure from 300 hPa to 1100 hPa with ±1 hPa precision. Additionally, its integrated metal-oxide (MOx) sensor reports volatile organic compound (VOC) levels in arbitrary units that correlate to total VOC concentration. Together, the AS7265x and BME688 form a multimodal sensing platform. While the AS7265x provides discrete spectral information enhanced by machine learning-based reconstruction, the BME688 ensures that spectral variations can be interpreted in relation to concurrent environmental conditions. The electronic subsystem of the prototype is built around an open-source SparkFun Thing Plus ESP32-S2 development board [59]. This module integrates a 32-bit Tensilica Xtensa LX7 core running at 240 MHz, with 320 kB of SRAM and 4 MB of external flash memory, providing sufficient resources for embedded preprocessing tasks and wireless communication. The board operates at 3.3 V and includes an on-board voltage regulator, simplifying integration with peripheral sensors. Typical active current consumption is on the order of 80–100 mA, allowing operation from USB power or portable battery packs. In addition to processing capabilities, the ESP32-S2 features a full 2.4 GHz Wi-Fi transceiver, which is used in this work for transmitting spectral and environmental data to a cloud server via MQTT and HTTP protocols.

### 2.2 Electronic integration

**Fig. 1:**
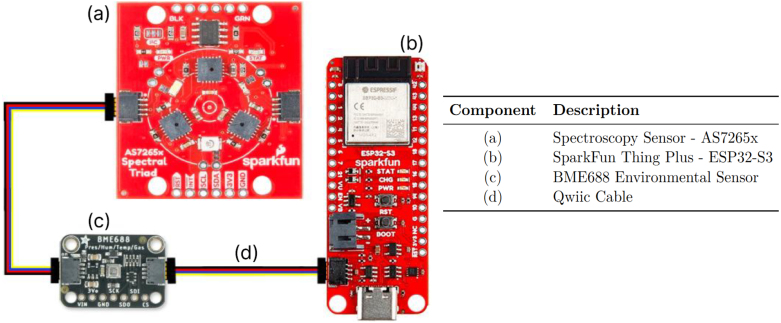
Electrical schematic illustrating the I${}^{2}$C-based connections between the SparkFun Thing Plus – ESP32-S2 and the AS7265x multispectral sensor and BME688 environmental sensor.

The electrical integration of detection modules is intended to enhance simplicity, robustness, and reproducibility. The AS7265x multispectral array and BME688 environmental sensor communicate via a shared I${}^{2}$C interface at 400 kHz. Using I${}^{2}$C simplifies wiring and allows for easy extension of sensors, making the system flexible and adaptable to various deployment scenarios. Each device is provided with a distinct bus address, which allows the ESP32-S2 microcontroller to manage them independently and without interference. To achieve synchronized acquisition, the firmware schedules sensor queries in a predetermined order, ensuring that spectral and environmental measurements occur in the same time frame. The microcontroller converts raw data into structured payloads by performing light preprocessing procedures such as normalization and local correction. These methods reduce variability and communication overhead while ensuring data integrity before transmission to the cloud. Next, the processed payloads are transmitted via the Wi-Fi transceiver integrated into the ESP32-S2. MQTT protocol is used for lightweight telemetry streams, while HTTP requests are used for structured data logging and storage. This dual-protocol implementation provides resilience against communication failures and enables compatibility with a wide range of IoT infrastructures. Fig. 1 shows the system wiring layout and illustrates the I${}^{2}$C connections between the ESP32-S2 development board, the AS7265x multispectral sensor, and the BME688 environmental sensor.

### 2.3 Mechanical architecture

A wide variety of custom farming tools and agricultural instruments have been 3D printed in the past [60], [61] and here the sensor module is housed in a custom enclosure fabricated by 3D printing with ASA filament. ASA was selected over alternatives such as polylactic acid (PLA) and acrylonitrile butadiene styrene (ABS) because it offers superior thermal stability, chemical compatibility [62], higher resistance to ultraviolet (UV) radiation, and greater long-term durability in agricultural and greenhouse settings, where prolonged exposure to light and temperature fluctuations can rapidly degrade materials and accelerate prototype aging [63].

The enclosure was designed to form a sealed optical chamber, guaranteeing that no stray light reaches the sensor and causes distortions in spectral measurements. This ensures that only radiation entering through the defined optical path contributes to the measurement, improving repeatability and reducing external interference. Similarly, this housing was designed to make the system compact and easy to assemble. The CAD design includes integrated spacer posts, screw protrusions, and alignment features to simplify sensor card assembly. It also provides a dedicated space for the optical diffuser and environmental sensing modules. The enclosure was 3D-printed using ASA filament. The main specifications of the 3D printer used in the fabrication process are summarized in Table 2, while the material consumption per part (ASA) is presented in Table 3. This modularity allows users to readily replace or rearrange individual components without altering the overall geometry of the housing. In addition, the enclosure maintains a fixed internal geometry, ensuring consistent alignment between the optical diffuser and the sensing element. This is particularly relevant given that deviations from an ideal cosine response are a known source of uncertainty in radiometric measurements [64]. By constraining the optical path and sensor orientation, the system minimizes variability associated with incident angle dependence. To fit the housing aperture, a 1.57 mm thick PTFE (Teflon) cosine-correcting diffuser measuring 26.20 × 32.61 mm was laser cut. PTFE was selected due to its strong optical transmittance in the visible and near-infrared regions, as well as its ability to approximate a Lambertian response, which ensures consistent angular sensitivity and reduces angular measurement bias. Nonetheless, it should be noted that the cosine-correcting behavior of the PTFE diffuser approximates, but does not fully eliminate, angular measurement uncertainty. Residual deviations from an ideal cosine response – particularly at incidence angles beyond 60° – may introduce systematic errors in the integrated band values [3], [64]. Furthermore, the system was validated at a fixed measurement distance of 350 mm from the LED source, as described in Section 7. Variations in mounting distance will affect the irradiance level received by the sensor, and users should maintain a consistent and documented distance for reproducible measurements. For deployment in CEA environments, it is recommended to mount the sensor facing upward at canopy level, directly below the luminaire, to minimize off-axis incidence and ensure consistency with the calibration geometry. The diffuser is mechanically coupled to the AS7265x multispectral sensor board (25 × 20 mm PCB). The board is mounted in a fixed plane relative to the diffuser using nylon spacers and M2 screws (length 8.97 mm), which provide mechanical stability, electrical isolation, and consistent optical alignment.

**Fig. 2:**
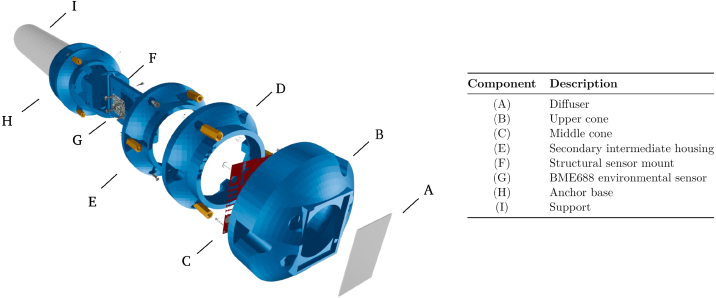
Exploded isometric view of the monitoring tower prototype, illustrating the cosine-corrector diffuser, the three-piece cone housing for the AS7265x multispectral sensor and BME688 environmental sensor, and the other mechanical parts.

Fig. 2 presents an exploded isometric view of the sensing tower prototype. The assembly follows a modular sequence: (A) cosine-corrector diffuser; (B–E) conical and cylindrical housing sections that form the light-sealed chamber; (F) structural mount securing the AS7265x sensor; (G) lateral placement of the BME688 environmental module, which remains exposed to airflow while isolated from the optical path; and (H–I) anchor base and rear support, which provide rigidity and mounting interfaces for external structures. This modular design allows components to be replaced or modified without affecting the overall geometry.

**Table 2:** 3D printing parameters for the optical enclosure.

Parameter	Value
Printer model	Creality K1 Max
Filament material	ASA (Acrylonitrile Styrene Acrylate)
Filament diameter	1.75 mm
Layer height Line Width	0.20 mm
Printing temperature	260 °C
Build Plate temperature	100 °C
Infill density	100% (rectilinear pattern)
Infill Pattern	Grid
Print speed	200 mm/s
Support structures	Tree
Support Pattern	Grid
Support Density	8%
Regular Fan Speed	90%

**Table 3:** Printing time and filament consumption for each component of the optical enclosure using ASA filament.

Part name	Printing time	Filament (g)
01_Upper cone	22 min 55 s	12.33
02_middle cone	47 min 17 s	21.35
03_light_sensor_holder	26 min 21 s	16.52
04_lower_cone02	1 h 18 min	44.41
05_upper_cone	1 h 05 min	34.27
10_Structural sensor mount	1 h 21 min	34.52

### 2.4 System overview

**Fig. 3:**
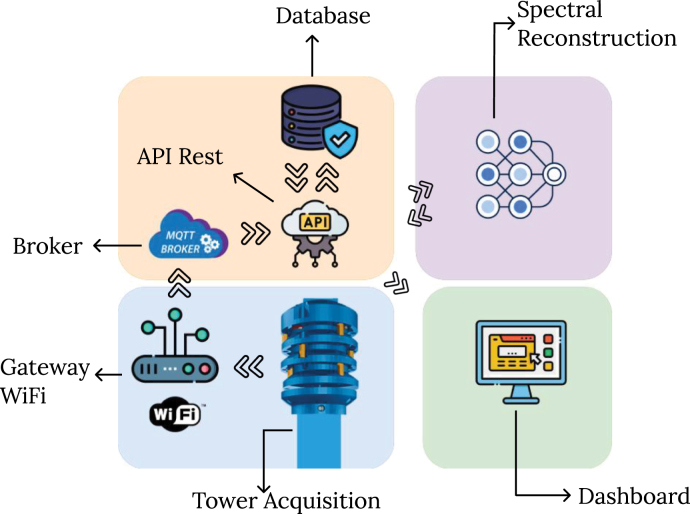
IoT architecture.

The device operates within a hybrid edge–cloud architecture that integrates local preprocessing with cloud-based spectral reconstruction. As summarized in Fig. 3 (IoT Architecture), the workflow is organized into four main axes:

1. On the edge, the ESP32-S2 performs a lightweight correction step with a TinyML Multilayer Perceptron (MLP) [27], [65]. Trained against the reference spectroradiometer (Hoppocolor OHSP-350C) within 410–780 nm, [66] this model reduces sensor-specific biases and nonlinearities in the AS7265x readings and outputs a 14-element adjusted vector aligned with the spectroradiometer’s calibrated range. The MLP is quantized to int8 for real-time on-device inference, with a .tflite footprint of roughly 12–20 kB, so measurements are corrected before transmission without noticeable latency.
2. The adjusted vector and the environmental telemetry are transmitted via the ESP32-S2’s Wi-Fi using HTTP for structured logging and MQTT for lightweight telemetry, as depicted in the Gateway/Broker communication blocks of Fig. 3. This dual-protocol approach improves resilience and compatibility with common IoT backends.
3. The HTTP endpoint is implemented as a FastAPI service, where a 1D Convolutional Neural Network reconstructs a dense, normalized SPD at 1-nm spacing (411 wavelengths from 410 to 780 nm). Trained on a mixture of real and synthetic spectra, the CNN-1D learns spectral priors that help recover features not directly resolved by the 20 nm FWHM Fabry–Pérot channels—mitigating the hardware’s resolution limits while keeping compute in the cloud and enabling over-the-air updates.
4. Reconstructed spectra and metadata are stored in MongoDB for traceability and querying. A web UI (React + Tailwind) displays the SPD and environmental variables in real time; the dashboard refreshes every 10 s, uses WebSockets for live updates, and exposes a REST API for historical queries.

To provide environmental context, a BME688 sensor logs temperature, relative humidity, and barometric pressure alongside the spectral data. The device packages measurements and metadata, transmits them to the backend for storage and visualization, and supports continuous monitoring in greenhouses and vertical farming systems.

### 2.5 Edge processing

At the edge, the ESP32-S2 manages asynchronous data acquisition and preprocessing tasks. The firmware first performs connectivity checks with the attached sensors. Once the AS7265x multispectral sensor is detected, a measurement is triggered using an integration time of 50 cycles and a gain of 24×. The raw data are then processed locally with a correction model based on a pre-trained MLP implemented through TinyML, which performs nonlinear compensation and normalization of the discrete spectral responses provided by the sensor. The MLP architecture consists of two hidden layers with 32 and 16 neurons, respectively, both using ReLU activation, and an output layer of 14 neurons with linear activation. The model was quantized to 8-bit integers (int8) to minimize computational load and inference latency, with a total of 1374 parameters (1312 int8 weights and 248 bytes of int32 biases), corresponding to approximately 1.52 KB of parameters. The resulting .tflite file occupies about 12–20 KB, making it suitable for deployment on a resource-constrained ESP32-S2 microcontroller.

After spectral correction, the system acquires environmental variables – temperature, humidity, and gas concentration – from the BME688 sensor. All measurements are serialized into JSON objects and transmitted to the backend through HTTP POST requests. Two payloads are generated per cycle: one containing the corrected spectral data from the AS7265x and another containing the environmental data from the BME688. Each payload includes a header with the device identifier to enable traceability.

The system operates with a transmission interval of two minutes, resulting in 720 cycles per day. With an average size of 1060 bytes per cycle, the daily data volume amounts to approximately 0.75 MB, making the device suitable for continuous long-term monitoring with modest network and storage requirements.

### 2.6 Backend and cloud processing

**Fig. 4:**
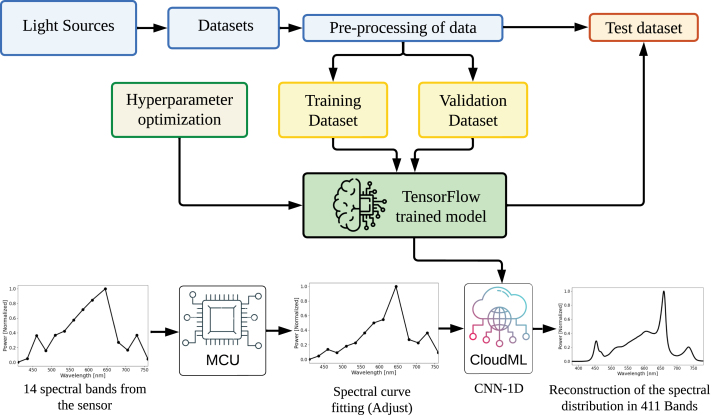
Workflow diagram showing the training and inference stages of the spectral reconstruction system.

Fig. 4 illustrates the complete pipeline for spectral correction and high-resolution reconstruction. The upper section depicts the training workflow, where spectral data generated from diverse light sources are preprocessed and divided into training, validation, and test sets. Hyperparameter optimization, implemented with the Optuna framework, is then used to guide the training of both the embedded MLP and the cloud-based CNN-1D models using TensorFlow. The lower section represents the inference stage of the deployed system.

In real-time operation, inference begins with fourteen spectral bands acquired from the AS7265x sensor. These measurements are normalized and corrected locally by a TinyML-optimized MLP running on the ESP32-S2 microcontroller, and the compensated spectral vector is then transmitted to a FastAPI-based cloud service. Although edge-side correction improves the sensor response, the AS7265x still provides only a sparse sampling of the underlying spectral power distribution, which is insufficient to fully resolve narrow horticultural LED peaks, mixed blue–red spectral profiles, and finer wavelength-dependent variations relevant to CEA. To address this limitation, a standard one-dimensional convolutional neural network (CNN-1D) was adopted to reconstruct a dense normalized SPD at 1 nm resolution over the 410–780 nm range. This architecture is well-suited to the problem because it formulates reconstruction as a one-dimensional regression task, efficiently learning local wavelength dependencies and spectral continuity from the corrected low-dimensional input while remaining compact, stable, and practical for deployment in the proposed edge–cloud monitoring system.

The CNN-1D was trained on a dataset of 10,000 spectra generated from combinations of horticultural LED emission profiles and physically inspired spectral models, including variations in intensity, peak wavelength shifts, and multi-source mixing. The dataset was specifically designed to emulate realistic lighting conditions encountered in CEA, capturing the spectral characteristics of common horticultural lighting systems. This approach enables the model to learn consistent spectral mappings within the distribution of interest while maintaining robustness to typical variations observed in practical deployments.

The network architecture consists of a single 1D convolutional layer with 104 filters, a kernel size of 9, ReLU activation, and a stride of 3, followed by a max pooling layer with a pool size of 2. A fully connected output layer with linear activation maps the extracted features to a 411-dimensional output vector representing the reconstructed spectrum. The model was trained using a batch size of 8, a learning rate of 0.001 with exponential decay, and a dropout rate of 0.09 for regularization, enabling the inference of high-resolution spectral distributions from discrete sensor inputs.

Alternative spectral architectures were also considered in light of recent hyperspectral deep learning literature. Grouped or band-wise spectral convolutions improve spectral feature extraction in high-dimensional hyperspectral classification [67], while 2D-CNNs are effective when spectral-spatial information is jointly available [68], [69]. However, these approaches were developed for tasks with many more spectral bands and, often, spatially resolved inputs. In contrast, the present work addresses a low-dimensional inverse regression problem with only fourteen corrected input channels and a continuous 411-point spectral output. Under these conditions, aggressive grouping may fragment the limited spectral information, and 2D-CNNs offer fewer practical advantages when the input is a single corrected spectral vector. For this reason, a conventional CNN-1D was retained as the most suitable architecture for dense spectral reconstruction from sparse multispectral observations.

Similarly, end-to-end frameworks for joint denoising and classification have shown advantages over decoupled pipelines in high-dimensional hyperspectral tasks [70]. The two-stage design adopted here is better aligned with the constraints of the proposed edge-cloud system. In this setup, the ESP32-S2 runs a lightweight int8 TinyML model, whereas the more demanding spectral reconstruction is performed in the cloud. This separation reduces the computational burden on the embedded device, enables over-the-air updates, and preserves deployment flexibility, while more integrated correction-and-reconstruction strategies remain a promising direction for future work.

Reconstructed spectra, together with metadata such as id_sensor and time_stamp, are stored in a MongoDB database to enable efficient querying and long-term traceability. Overall, this hybrid architecture combines lightweight edge inference with cloud-based high-resolution spectral reconstruction, achieving a practical balance between scalability, low-latency operation, and remote model update capability.

## 3 Design files summary

Table 4 shows all digital assets needed for building, programming, and deploying the monitoring tower prototype. Such items include mechanical parts (STL), firmware and machine-learning code, datasets, and full-stack IoT dashboard components.

**Table 4:** Digital design assets for the monitoring tower prototype, including 3D-printable parts, firmware, ML pipelines, datasets, and IoT dashboard components.

Design filename	File type	License	Location
Upper cone	.stl	CERN-OHL-S v2	https://doi.org/10.17605/OSF.IO/UM62Q
Middle cone	.stl	CERN-OHL-S v2	https://doi.org/10.17605/OSF.IO/UM62Q
Structural sensor mount	.stl	CERN-OHL-S v2	https://doi.org/10.17605/OSF.IO/UM62Q
Anchor base	.stl	CERN-OHL-S v2	https://doi.org/10.17605/OSF.IO/UM62Q
firmware_weather_pipeline	directory	MIT/Apache-2.0	https://doi.org/10.17605/OSF.IO/UM62Q
spectral_reconstruction_pipeline	directory	MIT/Apache-2.0	https://doi.org/10.17605/OSF.IO/UM62Q
cnn1d_reconstruction	directory	MIT/Apache-2.0	https://doi.org/10.17605/OSF.IO/UM62Q
training_dataset	.csv/txt	MIT/Apache-2.0	https://doi.org/10.17605/OSF.IO/UM62Q
dashboard_backend	directory	MIT/Apache-2.0	https://doi.org/10.17605/OSF.IO/UM62Q
dashboard_frontend	directory	MIT/Apache-2.0	https://doi.org/10.17605/OSF.IO/UM62Q

## 4 Bill of materials summary

Table 5 summarizes the components required to build the spectral sensing device. Each entry lists the designator used in the CAD and schematic files, the component description, quantity, unit cost, total cost, supplier reference, and material type. The enclosure is fabricated from ASA filament using 3D printing; therefore, the table reports the consumed filament cost rather than a direct purchase cost for a commercial housing. The complete system can be reproduced for less than US$150.

**Table 5:** Bill of materials for the open-source embedded spectral sensing device.

Designator	Component	Qty	Unit cost	Total cost	Source of materials	Material type
U1	ESP32-S2 development board (SparkFun Thing Plus)	1	24.95	24.95	https://t.ly/dkV0X	Electronics
U2	AS7265x multispectral sensor module	1	59.95	59.95	https://t.ly/NIK0a	Electronics
U3	BME688 environmental sensor (T, RH, P, VOC)	1	19.95	19.95	https://t.ly/F1qP0	Electronics
CN	Qwiic JST cables	2	2.25	4.50	https://t.ly/bktfq	Cable
ASA	ASA filament (163.4 g consumed)	1	6.86	6.86	https://t.ly/mVJFR	3D printing material
HW1	Brass hex standoffs, M3 female–female, **10 mm**	14	0.71	∼9.93	https://t.ly/MQl2J	Hardware
HW2	Nylon hex standoffs, M3 female–female, **6–8 mm**	4	0.50	∼2.00	https://t.ly/vWuHM	Hardware
HW3	Stainless steel pan-head screws, M3 × 6 mm	24	0.12	∼3.00	https://bit.ly/4o5g1Dh	Hardware
HW4	Stainless steel hex nuts, M3	4	0.25	∼1.00	https://bit.ly/4gXV8r6	Hardware
OPT	PTFE sheet **300 × 300 × 1 mm** (laser-cut cosine-corrector diffuser 26.20 × 32.61 mm)	1	12.00	∼12.00	https://bit.ly/4h8qcoj	Optical material

## 5 Build instructions

The following section provides detailed step-by-step instructions to assemble the multispectral monitoring prototype. All required design files are available in the *Design files summary* (Section 3), while the corresponding components are listed in the *Bill of materials summary* (Section 4).

### 5.1 Assembly process

**Fig. 5:**
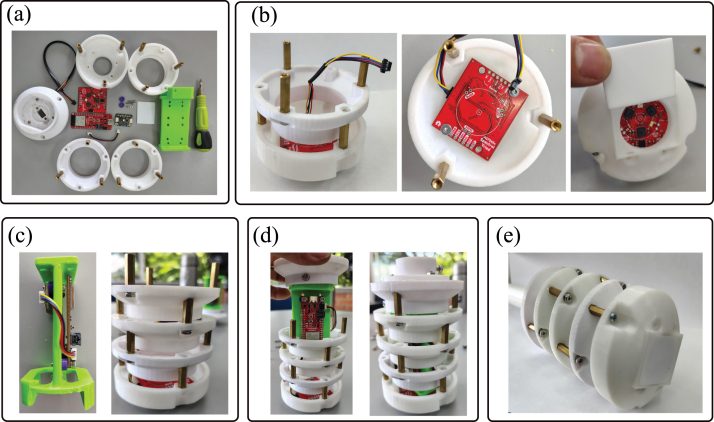
Assembly and setup of the spectral sensing prototype: (a) component layout before assembly, (b) diffuser and multispectral sensor placement, (c) lateral integration of the BME688 module, (d) ESP32-S2 installation, and (e) final assembled device.

The exploded isometric view in Fig. 2 and the wiring schematic in Fig. 1 should be consulted throughout the assembly process to ensure proper placement and orientation of the components. Additionally, representative photographs of the main steps are shown in Figs. 5(a–e).

1. Connect the AS7265x multispectral sensor (U2) and the BME688 environmental sensor (U3) to the ESP32-S2 Thing Plus development board (U1) using the two Qwiic JST cables as is depicted in Fig. 1. Both sensors share the same I^2^C bus (400 kHz) and are addressed independently (see Fig. 1). Ensure correct orientation of the connectors to avoid polarity errors. See also Fig. 5a for the components layout before assembly.
2. Print the enclosure parts (Upper cone.stl and Middle cone.stl) in ASA filament using fused filament fabrication 3D printing with a resolution of 0.2 mm and 50% infill. After printing, check the tolerances of the diffuser slot and screw holes. Light sanding may be required to guarantee a tight fit. Refer to Fig. 2 for the correct order of assembly and to Fig. 5a for the first fitting of the structure.
3. Place the PTFE cosine-corrector diffuser (A), cut to 26.20 × 32.61 mm and 1.57 mm thickness, into the aperture at the top of the housing. Below the diffuser, secure the AS7265x sensor (25 × 20 mm PCB) using nylon standoffs and M2 screws (8.97 mm length), as illustrated in Fig. 5b. This ensures alignment at the focal plane and provides both electrical insulation and mechanical stability.
4. Insert the BME688 sensor module laterally into its designated slot, allowing free airflow while keeping the optical chamber isolated. This placement ensures accurate readings of temperature, humidity, and VOC levels during operation, as shown in Fig. 5c.
5. Fix the ESP32-S2 board (U1) inside the lower housing section using the integrated standoff posts. Arrange the cables neatly to avoid blocking airflow or the optical path. Once the assembly is complete, the prototype should resemble the configuration in Figs. 5d and 5e.
6. Open the firmware project in PlatformIO. Configure the correct communication port in the platformio.ini file. The main code is in src/main.cpp, where the user must update the Wi-Fi SSID and password, the InfluxDB token, and the MQTT topic if required by the broker setup. Flash the firmware onto the ESP32-S2 and verify successful upload through the serial monitor.
7. Once the firmware is loaded, confirm Wi-Fi connection and data transmission by checking the InfluxDB server. A dedicated bucket (iot_rec) can be used to visualize the incoming records in real time. Alternatively, inspect the serial monitor to verify that payloads are being generated and transmitted.
8. Perform validation using a stable LED light source as a reference. Cover and uncover the diffuser to confirm that spectral values respond accordingly. Data can be exported from the database or the dashboard for visualization, allowing the spectral signature of the light source to be plotted and compared with expectations.

## 6 Operation instructions

This section describes the commissioning of the prototype and the assembly of the tower shown in Fig. 5(a)–(e).

In view (a), the preparation of the optical assembly is shown: the parts printed in ASA (upper and intermediate cones) are assembled around the PTFE cosine-corrector diffuser, which homogenizes the incident light and seals the optical chamber to prevent stray light.

View (b) illustrates the mounting of the AS7265x to the internal structural bracket, aligned with the diffuser to maintain the focal plane. The sensor board is fastened using nylon standoffs and M2 screws, and connected via Qwiic/I${}^{2}$C (400 kHz) to the ESP32-S2, sharing the bus with the BME688 while using independent addresses for each device. This arrangement ensures mechanical stability, electrical isolation, and simple, reproducible wiring.

In view (c), the lateral placement of the BME688 on the central bracket is shown, allowing it to be exposed to airflow while remaining isolated from the optical chamber, so that temperature, humidity, pressure, and VOCs are recorded representatively during spectral acquisitions.

View (d) shows how the central bracket is inserted into the housing from the bottom and how the ESP32-S2 board is secured in the lower section using the integrated posts. At this point, the firmware is flashed (Wi-Fi SSID/password and server updates) with PlatformIO, the connection is verified on the serial monitor, and telemetry is checked by covering/uncovering the diffuser to observe the spectral response.

Finally, view (e) shows the fully assembled tower on its base and rear bracket, ready for deployment in a greenhouse or indoor cultivation. With the hardware closed and powered, the unit enters its normal operating cycle: on-edge correction, Wi-Fi transmission, and cloud-based spectral reconstruction, with storage and visualization on the web dashboard for continuous monitoring.

The device operates in two main phases: (1) local spectral correction performed on the ESP32-S2 using a pre-trained regression model, and (2) remote spectral reconstruction with a CNN-1D model hosted in the cloud. In each acquisition cycle, the system transmits the corrected spectral channels together with environmental variables (temperature, relative humidity, barometric pressure, and VOC index) measured by the BME688. All data are packed into JSON payloads and forwarded to the backend for storage and visualization. The software is divided into two components: the embedded firmware running on the microcontroller and the cloud-based backend.

To deploy the firmware, download the repository edge_repository, open the platformio.ini file, and configure the correct serial port. In src/main.cpp, update the Wi-Fi credentials (ssid and password) and specify the IP address or DNS of the target server in the server variable. The default HTTP port is 3000, although this may be modified if the backend is hosted externally.

Once compiled and uploaded, the system can be tested with the following steps:

1. Check the serial monitor to confirm Wi-Fi connection. To carry out this task, open the serial monitor and confirm a successful connection. The message connection done will appear when the device is online. If the network is unavailable, the device will automatically retry, printing progress dots (.).
2. Verify the printed payloads. Covering the diffuser should result in spectral values near zero; uncovering it should produce measurable floating-point values that vary with the light source.
3. Ensure the data is being sent to the cloud server. In this case, it is necessary to confirm that the data are reaching the server by inspecting the corresponding bucket or dashboard. If transmission fails, check the error codes in Table 6 for guidance.

Errors −1 and −2 are the most common during initial deployment and usually indicate that the backend is inactive or unreachable. On the other hand, Error −4 suggests a malformed payload, which can occur if the JSON structure in the AS7265xModule is modified incorrectly.

**Table 6:** Error codes returned by the HTTPClient library.

Code	Description
−1	Connection error: failed to establish a link with the server
−2	Timeout or error while reading the server response
−3	Failure while sending data in a POST request
−4	HTTP encoding error caused by malformed payloads
−5	Invalid server response: unable to parse received data

## 7 Validation and characterization

**Fig. 6:**
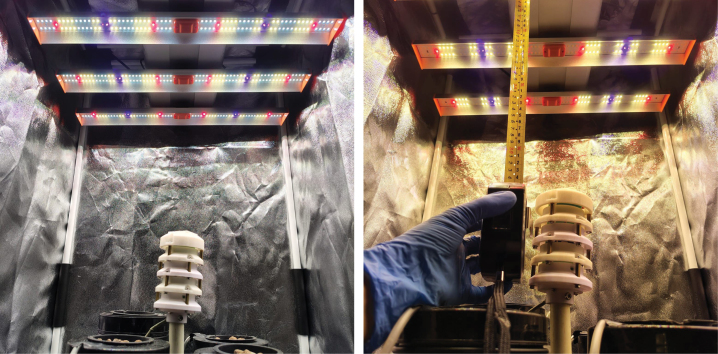
Assembled prototype of the multispectral sensing system for indoor crop monitoring.

The multispectral sensing unit was assembled following the steps outlined in Section 5 and integrated into the custom 3D-printed enclosure. Fig. 6 presents two photographs of the final prototype in operation. The image on the left shows the device positioned at canopy level within the indoor cultivation setup. The image on the right provides a top view of the configuration, highlighting the placement of the sensor at 350 mm below the artificial LED lamp used for plant illumination. This arrangement demonstrates how the sensing unit can be deployed directly within the cultivation environment without disturbing the light distribution. By operating *in situ*, the system captures not only the SPD of the lighting but also the associated environmental variables – temperature, humidity, and barometric pressure – recorded by the onboard sensors. The compact footprint and lightweight design make the device easy to install and relocate within experimental plots, enabling continuous monitoring under realistic growing conditions.

### 7.1 BME688 and AS7265x verification

To validate the performance of the proposed prototype, a controlled experiment was conducted using a SunPlus Sundro S250 LED light source [71]. During the experiment, a total of 16 measurements were collected at five attenuation levels to evaluate the performance of the AS7265x multispectral sensor and the BME688 environmental sensor. The objectives of the experiment were to compare the spectral response of the AS7265x with a high-resolution reference; and, secondly, to evaluate the improvements achieved by local correction in the microcontroller (MLP) and cloud-based reconstruction (CNN-1D).

**Fig. 7:**
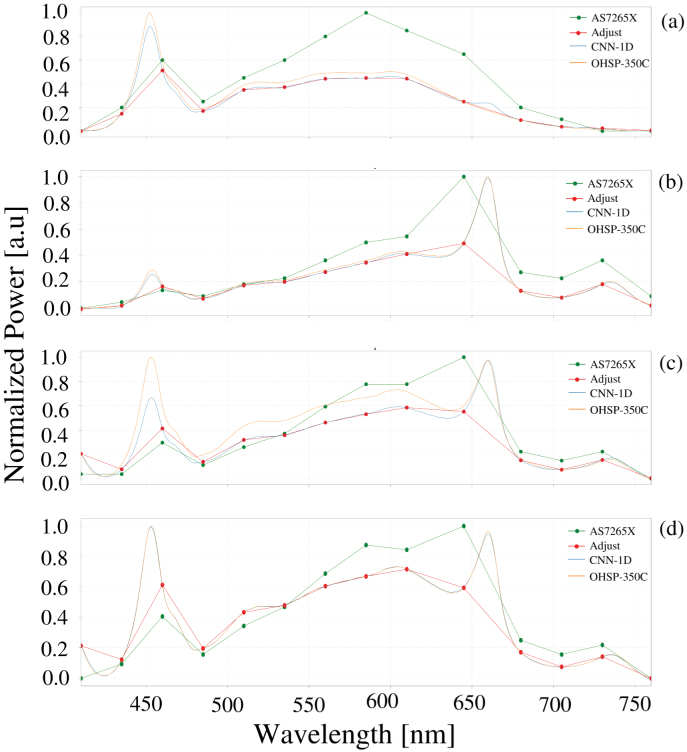
Spectral measurements under controlled LED illumination: (a) blue spectrum at 450 nm, (b) red spectrum at 660 nm, (c) mixed blue and red with higher intensity in red, and (d) mixed blue and red with comparable intensity.

Fig. 7 shows four representative measurements under different spectral conditions. Each subplot compares the raw AS7265x readings (green), the locally corrected output from the embedded MLP (red), the high-resolution reconstruction from the CNN-1D model (blue), and the reference spectrum from the OHSP-350C spectroradiometer (orange). Specifically, subplot (a) corresponds to a spectrum with a dominant wavelength around 450 nm (blue), (b) to a spectrum with a dominant wavelength near 660 nm (red), (c) to a mixed blue–red spectrum with higher intensity in the red region, and (d) to a balanced mixture of both wavelengths with comparable intensities.

The results reveal systematic deviations in the raw response of the AS7265x, especially around the emission peaks, due to the sensor’s 20 nm FWHM, which limits its spectral resolution. The integrated MLP correction stage, implemented through TinyML, significantly mitigates these discrepancies, reducing the root mean squared error (RMSE) from 0.183 to 0.035 and improving the coefficient of determination ($R^{2}$) from 0.72 to 0.95 on the validation set. Building upon this correction, the subsequent CNN-1D reconstruction achieved a global RMSE of 0.0135 and an $R^{2}$ of 0.98, yielding a dense normalized spectral output that closely follows the reference spectroradiometer across the entire range, capturing not only peak positions but also relative intensities.

The band-integrated error enables quantifying the model’s ability to preserve the spectral distribution within photobiologically relevant regions. In particular, the blue (400–500 nm), red (600–700 nm), and far-red (700–750 nm) bands are associated with key processes in CEA, such as photosynthesis and growth regulation. These bands fall within or extend the Photosynthetically Active Radiation (PAR) region, which is critical for plant development. The relative band-integrated error is defined as:

(1) $$E_{\text{band}}=\frac{\left|\int_{\lambda_{1}}^{\lambda_{2}}S_{\text{ref}}\left(\lambda \right)\;d\lambda -\int_{\lambda_{1}}^{\lambda_{2}}S_{\text{pred}}\left(\lambda \right)\;d\lambda \right|}{\int_{\lambda_{1}}^{\lambda_{2}}S_{\text{ref}}\left(\lambda \right)\;d\lambda }$$

As observed in Fig. 7, the main discrepancies are concentrated around the emission peaks, especially in the blue and red regions. The quantitative results in Table 7 confirm that the CNN-1D reconstruction maintains low relative errors within the blue and red bands across all evaluated cases, indicating that the model effectively preserves spectral energy in the regions of greatest photobiological relevance. However, cases (3) and (9) exhibit elevated far-red errors of 28.65% and 28.85%, respectively. This is explained by the low absolute energy present in the far-red region (700–750 nm) for these particular spectra: when the reference SPD carries negligible far-red content, small absolute reconstruction deviations produce disproportionately large relative errors. This is a known artifact of relative error metrics in low-energy spectral regions and does not reflect a systematic failure of the model in the far-red band [32]. Importantly, for cases where far-red radiation is the dominant or intended component of the illumination spectrum – as in photomorphogenic applications requiring explicit far-red control – the model should be validated independently with appropriate training data covering that spectral region.

Additionally, although PPFD is a widely used application-oriented metric, it was not explicitly computed in this work. However, since PPFD is directly derived from the spectral distribution within the PAR region (400–700 nm), the reported band-integrated errors provide an indirect but meaningful assessment of the model’s performance in this context.

**Fig. 8:**
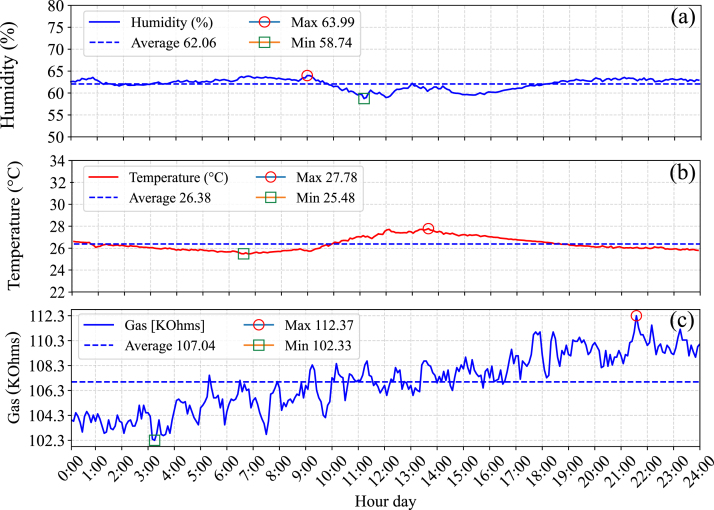
Environmental variables recorded by the BME688 during a 24-hour indoor monitoring period: (a) temperature, (b) relative humidity, and (c) gas resistance.

The BME688 sensor was integrated into the system to monitor ambient environmental conditions during spectral measurements. As detailed in Section 2, a basic calibration step was performed to ensure reliable operation under indoor conditions. Fig. 8 summarizes three representative environmental variables: temperature (a), relative humidity (b), and gas resistance (c). Although temperature and humidity tend to remain relatively stable in controlled indoor environments, the sensor captured subtle but relevant dynamics throughout a typical 24-hour cycle. The temperature ranged between 25.48 °C (recorded at 06:33) and 27.78 °C (at 13:38), with an average of 26.38 °C. Relative humidity followed a complementary trend, varying between 58.74% (at 11:13) and 63.99% (at 09:03), with a mean value of 62.06%. Gas resistance exhibited larger fluctuations during the day, ranging from 102.33 kΩ to 112.37 kΩ, with an average of 107.04 kΩ. The observed increase in resistance during the evening hours (peaking around 22:00) may be attributed to changes in air circulation or occupancy. These moderate diurnal shifts confirm that, even under controlled indoor conditions, the system is sensitive to environmental dynamics that could influence spectral measurements.

**Table 7:** Relative band-integrated error for selected representative spectral cases.

Case	Blue (400–500 nm)	Red (600–700 nm)	Far-red (700–750 nm)
(3)	3.15	1.25	28.65
(5)	11.10	1.70	2.05
(9)	0.27	0.27	28.85
(11)	2.23	3.49	7.00

Thus, these results validate the BME688’s capability to detect small but meaningful environmental changes, which are critical for contextualizing spectral measurements and ensuring proper interpretation during real-world deployments.

### 7.2 Cloud server deployment

The cloud server can be deployed on any virtual machine running a supported operating system. This includes infrastructure such as AWS, Azure, local datacenters, or even low-cost platforms like the ESP32. In this work, the process was validated on Ubuntu 22.04.5 LTS (Jammy Jellyfish), server edition [72].

An automated script included in the repository handles the installation. To deploy the server:

1. Clone the server repository into your target system.
2. Grant execution permissions to the deployment script: chmod +x server_deployment.sh
3. Run the script: ./server_deployment.sh

**Fig. 9:**
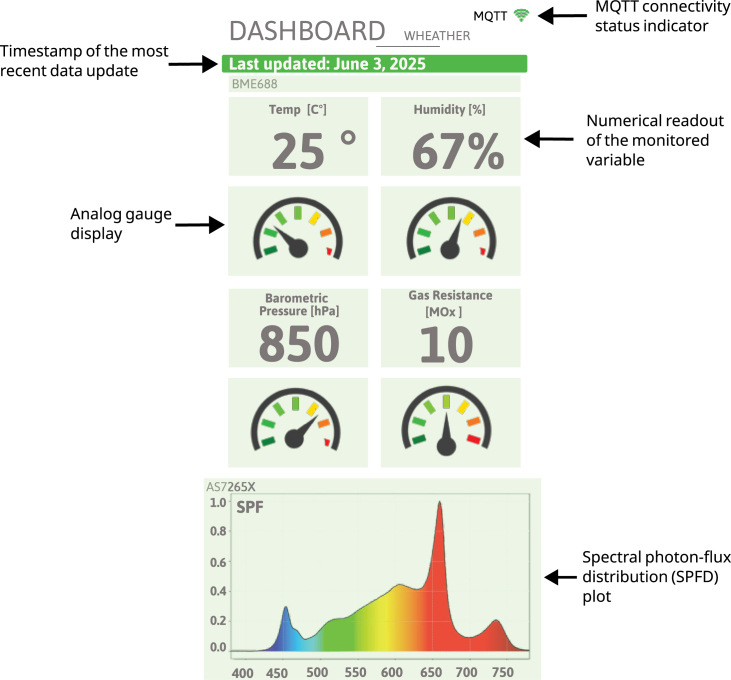
Web dashboard displaying BME688 environmental readings and the reconstructed SPD from the AS7265x.

The script installs all required dependencies and launches the backend server inside Docker containers. Upon successful completion, the message server done will appear on the terminal. If errors occur, verify that the repository was cloned correctly and that the script is executed from the appropriate directory. Once the server is running, return to the microcontroller’s serial monitor to confirm communication. A status code 205 indicates that the data has been accepted by the cloud server. To access the user interface, open a web browser and navigate to the server IP and port 8080, using the format: http://<server_ip>:8080. This will launch the system dashboard, which automatically refreshes every 10 s with the most recent sensor data. As shown in Fig. 9, the user interface was developed using React and Tailwind CSS, offering a clean and responsive layout. It provides real-time visualization through WebSockets and supports historical queries via a REST API.

The dashboard displays both environmental and spectral data in a highly intuitive format. Key features include:

• A timestamp indicating the most recent data update.
• MQTT connectivity status icon.
• Numerical readouts for temperature, humidity, barometric pressure, and gas resistance (MOx), each paired with an analog-style gauge for visual feedback.
• A dynamic SPD plot rendered from the reconstructed spectrum.

This compact and informative interface facilitates real-time monitoring of environmental and spectral conditions, making it particularly useful for indoor agriculture and lighting analysis scenarios.

## 8 Limitations and future work

Although the proposed device demonstrates clear advantages over comparable open-hardware systems in terms of spectral coverage, environmental integration, and reconstruction accuracy [3], [28], [29], [31], several limitations remain that should be considered for deployment and future development. Unlike systems limited to a small number of spectral channels or lacking environmental integration, the present design incorporates an 18-band AS7265x array covering the visible to near-infrared range, together with a BME688 module providing contextual measurements of temperature, humidity, pressure, and volatile organic compounds. The hybrid processing architecture – featuring lightweight correction via an embedded MLP and high-resolution reconstruction through a cloud-based 1D-CNN – overcomes the resolution constraints inherent to discrete-channel sensors, achieving results comparable to laboratory-grade instruments.

The spectral reconstruction model is inherently data-driven, and its performance is conditioned by the representativeness of the training dataset. The system was specifically optimized for the spectral distributions of discrete-peak horticultural LEDs commonly deployed in CEA. Consequently, its applicability is explicitly constrained to this domain: light sources with significantly different spectral characteristics – including broadband phosphor-converted white LEDs, multi-peak composite sources with overlapping emission bands, or aged and degraded LEDs whose spectral output has shifted from nominal – fall outside the validated scope of the model. For such sources, reconstruction accuracy may be substantially reduced, and independent validation with appropriate training data would be required before deployment.

The system’s radiometric accuracy is also subject to constraints imposed by the measurement geometry. The sensor was calibrated and validated at a fixed distance of 350 mm below the light source with the diffuser oriented upward. Deviations from this geometry – including changes in measurement distance, tilted mounting, or off-axis illumination angles – introduce additional uncertainty that has not been characterized in this work. Users deploying the system under different geometric configurations should perform an independent validation to quantify potential measurement bias associated with their specific setup.

As future work, we propose migrating the spectral reconstruction stage from the cloud to the edge by optimizing the CNN-1D model for execution directly on the microcontroller. This transition would reduce dependence on internet connectivity, increase system autonomy in remote agricultural environments, and strengthen the applicability of the device for continuous and real-time monitoring scenarios.

Additionally, future work should explore the incorporation of radiometric correction methods to improve the absolute accuracy of the reconstructed spectra. Techniques such as empirical line correction, sensor-specific response normalization, and scene-based calibration approaches have demonstrated effectiveness in improving spectral fidelity in related sensing systems [73], [74]. Integrating such methods into the processing pipeline could reduce systematic biases and extend the system’s suitability for applications requiring absolute irradiance quantification rather than relative spectral shape recovery.

## 9 Conclusions

The developed prototype demonstrates that it is possible to build a low-cost and fully open-source spectral system for controlled environment agriculture. By integrating multispectral and environmental sensors into a hybrid edge–cloud architecture, the design achieved a balance between portability, scalability, and ease of implementation, reducing the cost and complexity barriers of traditional laboratory spectroradiometers.

The use of a multilayer perceptron embedded in the microcontroller enabled the correction of nonlinearities and biases inherent to the AS7265x sensor, reducing the RMSE from 0.183 to 0.035. Building on this correction, the spectral reconstruction performed with a 1D-CNN model in the cloud reached an RMSE of 0.0135 and an $R^{2}$ of 0.98 compared to the reference spectroradiometer, confirming the ability of the two-stage approach to recover dense and reliable spectral distributions from affordable hardware under spectral conditions representative of horticultural LED lighting in CEA.

The inclusion of the BME688 sensor to record environmental variables (temperature, humidity, pressure, and gas concentration) provides contextual information for the spectral measurements and helps identify conditions that may affect the optical or electronic response of the system. This multimodal coupling, combined with the real-time web dashboard, demonstrates the practical utility of the device for agricultural monitoring, lighting optimization, and experimental analysis in indoor cultivation and vertical farming systems.

## CRediT authorship contribution statement

**Juan Morales-Guerra:** Writing – review & editing. **Juan Soto-Perdomo:** Writing – review & editing. **Juan Botero-Valencia:** Writing – review & editing. **Erick Reyes-Vera:** Writing – review & editing. **J.M. Pearce:** Writing – review & editing.

## Human and animal rights

In this work, no human or animal studies were conducted.

## Declaration of competing interest

The authors declare that they have no known competing financial interests or personal relationships that could have appeared to influence the work reported in this paper.
